# Investigation of Measles Outbreak among Thai and Migrant Workers in Two Factories in Nakhon Pathom, Thailand, 2019

**DOI:** 10.3390/ijerph17134627

**Published:** 2020-06-27

**Authors:** Suphanat Wongsanuphat, Phanthanee Thitichai, Rungrot Jaiyong, Patchanee Plernprom, Kanthika Thintip, Charuttaporn Jitpeera, Rapeepong Suphanchaimat

**Affiliations:** 1 Division of Epidemiology, Department of Disease Control, Ministry of Public Health and Thailand, Nonthaburi 11000, Thailand; suphanat.wong@gmail.com (S.W.); phanthanee@gmail.com (P.T.); rungrot00jaiyong@gmail.com (R.J.); patchanee.p08@gmail.com (P.P.); t.ganthiga@gmail.com (K.T.); charuttaporn@gmail.com (C.J.); 2 International Health Policy Program, Ministry of Public Health, Nonthaburi 11000, Thailand

**Keywords:** measles, migrant, outbreak, Thailand

## Abstract

On 22 March 2019 the Thai Department of Disease Control (DDC) was notified that 16 workers, including Thai and Myanmar migrant workers, from two factories located in Nakhon Phathom Province, had presented with a fever with rash during the previous 2 weeks. Active case finding was conducted among workers in both factories using face-to-face interviews. Suspected cases were defined as a worker who developed fever with rash with one of the following symptoms: cough, coryza or conjunctivitis. Testing for measles IgM antibodies and viral identification through throat swabs by polymerase chain reaction (PCR) were performed to confirm diagnosis. Vaccination history among cases was reviewed. Nationality and age-specific attack rates (AR) were calculated. An environmental study and a social network analysis were conducted to better understand the transmission process. A total 56 cases (AR = 0.97%) were identified. Of 21 serum measles IgM collected, 8 (38.0%) were positive. Of 8 throat swabs collected, 5 (62.5%) were positive for measles genotype *D8*. The disease attack rate in migrant employees was twice as large as the rate in Thai counterparts (AR = 0.7 and 1.4%). The first case was identified as a Myanmar worker who arrived in Thailand two weeks prior to his illness. The Myanmar workers’ accommodation was more crowded than that for Thai workers. The hot spots of transmission were found at a drinking water tank which had shared glasses. Among the cases, 62.5% could not recall their vaccination history, and 25% had never had an injection containing a measles vaccination. The majority of migrant cases had never completed a two-dose measles vaccination. To halt the outbreak, measles vaccines were administered to the employees, particularly those working in the same sections with the cases and shared glasses were removed. For future policy action, a vaccination program should be incorporated into the work permit issuance process.

## 1. Introduction

Measles is a highly communicable viral disease causing substantial morbidity and mortality globally. Cases are mostly in low- and middle-income countries with 6.7 million cases and 110,000 deaths estimated by the World Health Organization in 2017 [1]. The timing and the magnitude of measles epidemics are determined by seasonal fluctuations [2,3] and the degree of local susceptibility [4]. According to the report of the Thai Department of Disease Control (DDC), Ministry of Public Health (MOPH), the yearly volume of new measles infections was approximately 5000 nationwide [5]. In addition, more than 10% of measles cases were found in non-Thai citizens with approximately 70% in people from Myanmar. Reported case fatality ranged from 0.01 to 0.20% from 2014 to 2019 [6].

The expanded program of immunization (EPI) is the main policy to strengthen population immunity in Thailand and the measles-mumps-rubella (MMR) vaccine is included in the list of EPI. The first dose of MMR vaccine (MMR1) is provided to all children aged 9–12 months old and the second dose (MMR2) to those aged 2.5 years old [1]. The MMR1 and MMR2 vaccine coverage in Thailand in 2018 is approximately 99% and 87%, respectively.

Among the 1.3 million cross-border migrants holding work permits in Thailand in 2009, 82% were from Myanmar [7]. In Myanmar, national immunization coverage varies widely (from 38% to 93% in 2012 for MMR1) because of limited health infrastructure and funding as well as difficulty in accessing services [8]. A recent report in Myanmar in 2018 showed the prevalence of MMR1 and MMR2 to be about 93% and 87%, respectively [9]. This concern was raised in many policy dialogue spaces as migrant populations entering Thailand might not be vaccinated against MMR from the outset and this creates a risk of measles epidemic, not only among migrant communities but also among Thai populations [10].

On 22 March 2019, the Division of Epidemiology (DOE) of the Thai-DDC was notified by the Office of Diseases Prevention and Control Region 5 (ODPC 5) that 16 employees of both Thai and Myanmar origin in two factories (Factory-D and Factory-S) in Nakhon Phathom Province had presented with a fever with rash during the previous 2 weeks. This notification activated the Thai DDC to promptly investigate the cause of the outbreak and to provide proper recommendations to halt disease progression. The investigation took place between 22 and 29 March 2019 by the joint investigation team comprising staff from the DOE, the ODPC 5 and the local health office. The objectives of this study were to: (i) confirm the outbreak and diagnosis; (ii) describe the epidemiological characteristics of the outbreak; and (iii) identify possible causes of the outbreak and provide proper recommendations to control the outbreak.

## 2. Methods

### 2.1. Overview of the Study

A cross-sectional study was employed. To identify a measles case, a set of criteria was established. A suspected case of measles was defined as an employee in Factory-D or Factory-S with current or history of fever (≥38 °C) and rash with at least one of following symptoms: coryza, cough, conjunctivitis and Koplik’s spot, or who was diagnosed with measles by a physician between 14 February 2019–30 April 2019. A confirmed case was a suspected case who had a positive seroconversion of measles IgM or positive viral detection from a throat swab polymerase chain reaction (PCR). For active case finding, we measured body temperature and performed an individual face-to-face interview with a semi-structured questionnaire with all employees in every section of the factory which had reported measles cases. In addition, we asked the factory foreman to perform a verbal screening in all other sections and report to us if they found any worker with signs and symptoms that met our case definition.

### 2.2. Data Collection

A semi-structured questionnaire was used to identify whether an individual was classified as a case. Among the cases, the investigation team collected information about employees’ demographic details (age, sex and nationality), types of work, signs and symptoms, vaccination history, treatment, and risk behaviors (particularly, sharing glasses at the drinking fountains). On average, each participant took about five minutes to complete the questionnaire. In order to confirm diagnosis among the suspected cases, a throat swab for measles PCR was collected [11,12].

We randomly selected one third of suspected cases to test for measles IgM and IgG (serum), rubella IgM and IgG, (serum) and zika PCR (serum and urine) in order to confirm a measles diagnosis and rule out other possible diagnoses which have signs and symptoms close to measles [9,11]. All specimens were tested by the National Institute of Health (NIH), the Department of Medical Sciences, MOPH [12].

### 2.3. Data Analysis

Descriptive statistics were applied. The findings were shown in terms of percentage of cases among the population, also known as attack rate (AR). The age-specific, nationality-specific, factory-specific attack rates and nationality-specific vaccine coverage among cases were calculated. In addition, either a Chi square test or Fisher Exact test was performed to assess whether the difference in AR between groups of interest and vaccine coverage exhibited statistical significance (cut-off at *p* < 0.05).

To better understand the process and the dynamics of transmission during the outbreak, an exploratory social network analysis was performed. The analysis findings were displayed in a form of a sociogram that maps a personal contact and a geographical contact among the cases [13].

### 2.4. Environmental Survey

Apart from the data collection via questionnaire, the investigation team carried out a brief environmental study via a walk-through survey with direct observation on the physical structure of the factories (such as working zone and dining zone) and routine activities of the employees. This helped to assess if the factory layout and the employees’ behavior aligned with infection control measures as recommended by WHO. A brief interview with the factories’ manager was performed to validate the investigators’ understanding. The interview lasted about 30 min without audiotaping.

### 2.5. Ethics Clearance

As this study was conducted as part of a routine investigation of the DOE in response to the outbreak, it was not necessary to obtain ethics approval from the MOPH. However, in this study, the researcher strictly followed ethical standards in the research process, and all individual information was strictly kept confidential and not reported in the paper.

## 3. Results

### 3.1. Setting Overview

Factory-D and Factory-S were neighboring factories within the same parent company, located in Nakhon Pathom, an adjacent province of Bangkok. The working shifts were day shift (8 a.m.–8 p.m.) and night shift (8 p.m.–8 a.m.). There were a total of 5581 employees in both factories combined (2691 employees in Factory-D and 2890 employees in Factory-S). Almost two-thirds of the employees were Thai (3531 employees, 63.3%), while the remainder were Myanmar migrant workers (2051 employees, 36.7%).

### 3.2. Outbreak Description

The investigation team focused on the affected sections of both factories. With active case finding, a total of 2172 employees were screened (39.8% of all employees). The team identified 56 cases. Of these 56 cases, 47 (83.9%) were from Factory-S and 9 (16.1%) were from Factory-D. Thirteen individuals were identified as confirmed cases (23.2%) and 43 as suspected cases (76.8%). Approximately half of the cases (30 cases, 53.6%) were Myanmar migrant workers. Males constituted the majority of the cases (67.9%). Almost half of the cases were young adults aged 20–29 years (27 cases, 48.2%). The median age of the cases was 29 years (Q1–Q3: 25–35 years).

Of the 56 cases, 31 (55.4%) visited health care facilities. Most of them (27/31) were treated as outpatients, while only 4 cases were admitted to the nearby hospital. However, no complications or deaths were observed.

An overall AR among all employees was 1.0% (56/5581) but the AR rose to 2.6% (56/2172) if focused on those who were screened. The AR of factory-S was more than 5 times higher than that of factory-D (1.6% and 0.3%, respectively). The section-specific AR ranged from 0.3% to 3.9% and varied by working zones with statistical significance (Chi square = 42.5, *p* = 0.001) (Table 1).

Among Myanmar migrant workers, the AR was almost twice that of Thai workers (1.4% vs. 0.7%) (Chi-square = 6.8, *p* = 0.008). In addition, age-specific AR varied across age groups with statistical significance (Chi square = 68.4, *p* = 0.001)). Young adults (20–24 years) showed the greatest AR in comparison with other age groups (23 cases, 2.21%), Table 2.

**Figure 1:**
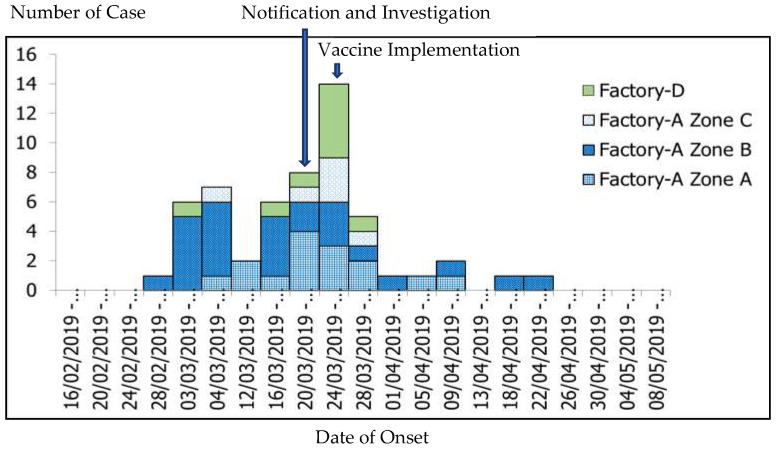
Epidemic curve of measles outbreak in Factory-S and Factory-D from 14 February to 28 March 2019 (*N* = 56).

The epidemic curve indicated a propagated source outbreak. The first case was a Myanmar migrant worker aged 27 years. He could not remember his measles vaccination history. He had just travelled back from Myanmar and started working in Factory-S, Section B on 11 February 2019. By late February, he developed fever, followed by maculopapular rashes. Then, the number of cases rapidly rose to 15 within a few days and cases spread to all sections in Factory-S. On 17 March 2019, the first two cases in Factory-D were reported. One of them had history of contact with her cousin who worked in Factory-S. Her cousin’s onset of symptoms started on 7 March 2019. They both shared the same accommodation, a private car, and utensils when dining every day. The last case of this outbreak was a worker at Factory-S which was reported on 25 April 2019 (Figure 1).

Regarding personal histories of measles vaccinations, 62.5% of the cases (35/56) could not recall any information, 25.0% (14/56) had never received a measles vaccine, 7.1% (4/56) had completed a two-dose and 5.4% (3/56) had received a one-dose vaccination course as recommended by the MOPH. Among the Myanmar cases, 19 of them (63.3%) could not recall their history of measles vaccination, and 11 of them (36.7%) never had their measles vaccination history. For the Thai cases, 7 had (26.9%) received at least 1 dose of measles vaccination, 3 (11.6%) had never received a measles vaccine and 16 (61.3%) could not recall. There was a significant difference between the Thai and Myanmar migrant worker cases vaccination history (Fisher Exact test, *p*-value = 0.003) (Table 3).

### 3.3. Laboratory Study

Of the 56 suspected cases, seventeen had their blood tested for measles IgM. Eight out of these seventeen (47.1%) were found positive for measles IgM. Furthermore, a throat swab was collected from eight individuals and five of them (62.5%) returned positive for measles (genotype *D8*). Neither zika nor rubella were detected from either urine or serum (Table 4).

### 3.4. Environmental Study

**Figure 2:**
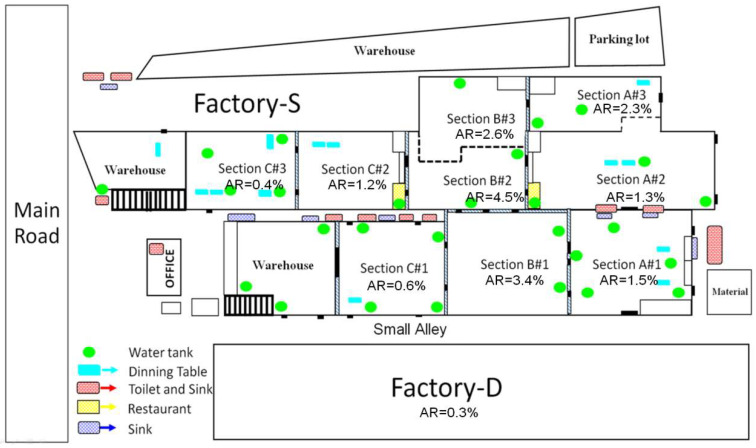
Diagram illustrating the physical structure of Factory-S and Factory-D and attack rate (AR) in each sub section.

Factory-S and Factory-D were separated from each other by a small alley. There were three major sections, namely, Section A, B, and C in Factory-S. Section B of Factory-S, the workplace of the index case with the highest attack rate, had an air-conditioned closed space. All other sections were open air zones with adequate air ventilation. Most employees were working in their own section, and only a few of them wandered to other zones during the working hours. Employees from diverse sections shared drinking fountains located in front of the restrooms and canteens. There were shared glasses at every drinking fountain. Hand washing basins were available in front of the toilets but neither soap nor hand washing gel was available. (Figure 2)

In terms of accommodation, the majority of employees stayed in dormitories or apartments located near the factories. The quality of accommodations varied depending on affordability by each individual. Thai and Myanmar migrant worker communities were separated. In general, the accommodation for Thai employees was cleaner, less overcrowded, and had better air ventilation.

### 3.5. Social Network Analysis

**Figure 3:**
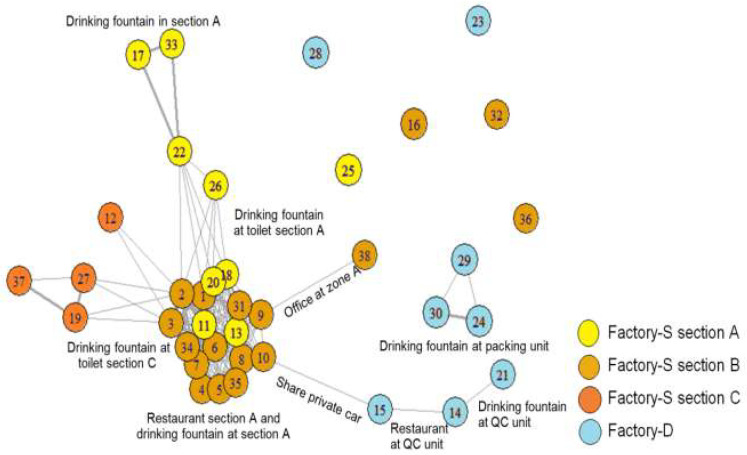
Sociogram illustrating the connection of cases (*N* = 38). Note: The figure inside a bubble refers to the ordering number of a case, counted by onset date. Different colors of the bubbles indicate different working sections. The thickness of a line indicates the strength of network. A thin line means either physical contact (friends or roommates) or geographical contact (working in the same workplace), while a thick line indicates that the persons that linked together had both types of contact.

Figure 3 presents a sociogram that elaborates the pattern of contact among the cases. Canteens and drinking fountains appeared to be hot spots where many employees came into contact. The first case of Factory-D made contact with the index case from Factory-S by their daily activity of sharing both a private car and accommodation.

## 4. Discussion

Overall, this study confirms the presence of a measles outbreak in two private factories in Nakhon Pathom, Thailand. However, identification of the first case is not conclusive. The genotypic finding from five specimens of throat swab showed genotype *D8*, which is currently a genotype circulating in Thailand and Myanmar [14]. A possibility that the first case acquired measles from Myanmar could not be excluded as the timing between date of entry to Thailand and onset of symptoms was still within the incubation period which is 7–23 days [15]. Further phylogenetic analysis is required to confirm the source of outbreak.

The transmission chain can be partly explained by results displayed in the sociogram. Most of the first generation transmission occurred in Factory A, section B, then it spread to Factory A, sections A and C. This might be due to indirect contact with infectious particles presenting on the glasses at the common fountain. The transmission chain from Factories A to B could be initiated from employee #10 (Factory A) and employee #15 (Factory B) who shared the same accommodation. Thus the spread from employee #10 and employee #15 could be due to either direct droplet transmission or indirect contact via fomites. The major route of transmission within a single factory section might be from both airborne transmission and indirect contact with infected surfaces such as shared glasses at the drinking fountains inside each factory section, while further spread of the disease from one factory section to another might be due to workers sharing glasses at the common drinking fountains. Without proper cleaning, measles can remain contagious in the air or on infected surfaces for up to two hours [16]. However, we could not collect specimens from the surfaces of shared glasses to confirm this possible route of transmission due to the limitation of laboratory testing.

The overall AR in this study is 1.1%, somewhat smaller than previous measles outbreaks in crowded areas elsewhere in Thailand, such as migrant worker camps and prisons (1.9%–25.9%) [17,18,19]. Such a difference might be explained by early case detection and isolation by the local health staff which took approximately three weeks since the onset of the first case compared with five weeks in previous outbreaks [20,21].

Interestingly, over half of cases in this outbreak are Myanmar migrant workers. The attack rate among Myanmar migrant workers is double the rate of that among Thai workers (1.4 % vs. 0.7%). This finding is likely explained by the following reasons. Firstly, vaccine coverage among Myanmar migrants living in Thailand was much lower compared with the native Thai populations [20]. The root cause of poor vaccine coverage in migrant communities is multifaceted, including language difficulties, financial barriers, and lack of awareness of healthcare rights and service availability [22]. In addition, even in Myanmar, national immunization coverage is quite low (especially in border areas) and is vastly diverse by regions (38–93% in 2012) [10]. Secondly, most accommodation in Myanmar migrant communities is overcrowded and suffers from poor sanitation and inadequate air ventilation relative to the Thai communities’ accommodation [23]. Overcrowded environments and inadequate sanitation always facilitate the transmission of the measles virus [24].

The median age in this outbreak is higher than in the previous outbreaks occurring in migrant worker camps and temporary shelters [18,25,26]. There is also a clear shift towards adult measles in Thailand (from 28.5% of cases in 2017 to 49% in 2019) [27]; this phenomenon reflects the fact that the measles situation in Thailand follows a bimodal distribution, similar to that of many other countries. [28]. Germany also faces the rise of measles cases in adults (from 11% of cases in 2003 to 43% in 2013). This was partly explained by the large influx of refugees from Eastern Europe [28]. Currently, like many developed countries, measles vaccines are offered free of charge to all Thai children and children of migrants who are a beneficiary of the Migrant Health Insurance Scheme (MHIS) managed by the MOPH [29,30]. However, for migrant workers coming into Thailand to seek jobs, there is no policy to assess if they already have immunity to certain diseases (including measles) before obtaining a work permit [31]. This gap in policy is among many contributory factors that create a risk of outbreak in both migrant communities and the Thai population.

To contain the outbreak, the team contacted the local health facilities to provide measles vaccines for the factories’ employees. A total of 1349 doses of measles vaccine were provided to employees, prioritized according to the attack rate of each section and age groups at risk. In addition, people that had a suspected measles case were given the measles vaccine as post-exposure prophylaxis (PEP) within 72 h in order to limit measles transmission [32].

Other supporting measures, apart from vaccination, are also required. A daily screening system was established by the factory foremen. If a suspected case arises, the foremen are obliged to inform the factory nurses and health staff at the nearby hospital. The factory managers were advised to remove shared glasses at the drinking fountains. All employees were encouraged to bring their own water flask and adhere to stringent hygiene practices (hand washing and avoiding sharing of personal belongings). The factories were advised to install new drinking fountains in at least one location for each section.

At the macro-level, recommendations are for the MOPH to work closely with the Ministry of Labour to launch a vaccination program and ensure vaccinations as part of a precondition to obtain a work permit for migrants who have never been vaccinated or those whose vaccination history is in doubt [31]. In addition, an adult immunization program for migrant workers should be considered alongside the existing childhood immunization program. This measure will provide huge benefits to both migrants and Thai citizens in terms of public health security. An electronic individual immunization registry should be established and this should be seamlessly linked with the immunization records in the country of origin of migrants [33]. The synchronized data platform will be of huge value in terms of outbreak investigation and control, and supporting nationwide academic activities in the future (such as vaccine effectiveness and vaccine coverage studies among Thais compared with migrants).

Regarding methodological limitations, the investigation team did not have enough time and resources to undertake a comprehensive screening of all employees in the factories. The focus was on the sections with notified cases. Some measures were implemented immediately to halt the outbreak without a need for the investigation to be completed across both entire factories. In addition, language barriers might have created misunderstandings on the information retrieved and this might have led to misclassification bias. Although local interpreters assisted the investigation, the quality of translation could not be fully assured. Furthermore, uncertain measles vaccination history was found in almost two-thirds of cases. This could have led to misclassification bias as well. In order to reduce or eliminate this problem, electronic individual immunization registry is recommended. Lastly, we did not collect data from a control group or consider other epidemiological factors such as economic factors in order to compare groups. Further studies are recommended to fill the gaps of this study.

## 5. Conclusions

This study confirms the advent of a measles outbreak (genotype *D8*) in two factories in one of the provinces in Central Thailand. The first case and half of all cases were Myanmar migrant workers. The disease attack rate among migrant employees was twice the rate among Thai counterparts. The majority of migrant cases had never completed a two-dose measles vaccination. To halt the outbreak, measles vaccines were administered to all employees, and shared glasses were removed. For future policy action, a vaccination program should be incorporated into the migrant work permit issuance process.

## Figures and Tables

**Table 1:** Number of total population, cases, and attack rate by working zones in Factory-S and Factory-D from 25 February 2019 to 29 March 2019 (*N* = 56).

Zone	Population (*N*)	Screen (*n*)	Percent Screening	Number of Case	AR by Population (%)	AR by Screening (%)
Factory-S	2890	1251	43.28	47	1.63	3.76
• Zone A	859	407	47.37	15	1.75	3.69
• Zone B	633	355	56.08	25	3.94	7.04
• Zone C	711	350	49.23	6	0.84	1.88
• Warehouse	189	139	73.54	1	0.53	0.79
Factory-D	2691	921	34.22	9	0.33	1.00
Total	5581	2172	39.80	56	1.00	2.58

Note: AR by Screening means incidence of cases in the screened population (number of cases/screened population) × 100.

**Table 2:** Number of total population, cases and attack rate by age groups in Factory-S and Factory-D from 25 February 2019 to 29 March 2019 (*N* = 56).

Age Group—Years	Population—*n*	Case—*n*	Attack Rate (%)
16–19	131	0	0
20–24	1039	23	2.21
25–29	1397	19	1.36
30–34	103	10	0.97
35–39	840	1	0.01
>39	643	2	0.03
Unknown		1	-
Total	5581	56	1.00

**Table 3:** Proportion of measles vaccination among Thai compared to migrant worker measles cases (*n* = 56).

	Thai	Migrant Worker	Total
No Measles Vaccination	3 (11.6%)	11 (36.7%)	14 (25.0%)
1 Dose of Measles Vaccination	3 (11.6%)	0 (0%)	3 (5.4%)
2 Doses of Measles Vaccination	4 (15.5%)	0 (0%)	4 (7.1%)
Can’t Remember	16 (61.3%)	19 (63.3%)	35(62.5%)
Total	26 (100%)	30 (100%)	56 (100%)

**Table 4:** Number of specimen samplings and the laboratory findings on measles, zika, and rubella.

Antigen	Laboratory Test	Specimen	Sample	Result—*n* (%)		
				Positive	Borderline	Negative
Measles and Rubella	IgM	Serum	17	8 (53%)	3	6
	PCR	TS+NPS	8	5 (genotype *D8*) (62%)	0	3
CR for Zika	PCR	Urine	9	0	0	9
	PCR	EDTABlood	4	0	0	4

Note: EDTA = ethylene diamine tetraacetic acid, NPS = nasopharyngeal swab; TS = throat swab, PCR = polymerase chain reaction.
